# Target mRNA inhibition by oligonucleotide drugs in man

**DOI:** 10.1093/nar/gks861

**Published:** 2012-09-18

**Authors:** Helen L. Lightfoot, Jonathan Hall

**Affiliations:** Department of Chemistry and Applied Biosciences, Institute of Pharmaceutical Sciences, ETH Zürich, CH-8093 Zürich, Switzerland

## Abstract

Oligonucleotide delivery *in vivo* is commonly seen as the principal hurdle to the successful development of oligonucleotide drugs. In an analysis of 26 oligonucleotide drugs recently evaluated in late-stage clinical trials we found that to date at least half have demonstrated suppression of the target mRNA and/or protein levels in the relevant cell types in man, including those present in liver, muscle, bone marrow, lung, blood and solid tumors. Overall, this strongly implies that the drugs are being delivered to the appropriate disease tissues. Strikingly we also found that the majority of the drug targets of the oligonucleotides lie outside of the drugable genome and represent new mechanisms of action not previously investigated in a clinical setting. Despite the high risk of failure of novel mechanisms of action in the clinic, a subset of the targets has been validated by the drugs. While not wishing to downplay the technical challenges of oligonucleotide delivery *in vivo*, here we demonstrate that target selection and validation are of equal importance for the success of this field.

## INTRODUCTION

Successful drug development is a precarious combination of the right target, the right drug, the right disease and the right patient population. When one of these parameters is ill-chosen the drug fails. As a handful of oligonucleotide drugs inch toward regulatory approval, consensus according to recent reviews and perspectives is that the ‘delivery problem’ is the pivotal factor which will see this new therapeutic paradigm stand or fall (1,2). However, hard evidence for the failed delivery of a drug *in vivo* is rarely available. While not wishing to downplay the technical challenges of oligonucleotide delivery *in vivo*, to our view it plays equal fiddle to a parameter that is rarely discussed, that of target validation.

The pharmaceutical industry considers the optimal drug target to have a well-understood role in the pathophysiology of the disease, to be disease-modifying when modulated with drugs, to be non-essential in unaffected tissues, to have its expression restricted to the disease tissue, to be assayable with a biomarker to monitor therapeutic efficacy and to have a favorable competitive situation (3). Drug targets can be classified according to their clinical validation (4). Validation of a target is the confirmation of its functional role in the disease pathology of patients. This is ultimately achieved using the drug in phase 2 clinical studies. An unvalidated target represents a new mechanism of action which has not previously been shown to have therapeutic utility in patients for a particular indication. Although new mechanisms of action offer the possibility of breakthrough treatments, the success rate of drug development involving unvalidated targets is lower than for validated targets (5). One analysis gave the probability of success (first dose in patients to approval) for a new mode of action as 9%, compared with 23% for a drug with an established mechanism (6). A second report put the value at 7% (small-molecule drugs; phase 1 to launch) (7). We have analysed oligonucleotide drugs which have been recently evaluated in clinical trials with respect to their target inhibition and their target validation. We consider downregulation of the target to be an indicator for successful delivery of the drug to the target tissue. We identified 26 oligonucleotide-target combinations which are, or were until recently, in phase 2 clinical status or above (Table 1) according to Thomson Reuters Integrity database. The analysis shows that the targets of these drugs represent new mechanisms of action and therefore should be considered high risk. Most of them fall outside of the drugable genome and thereby avoid competition from small-molecule drugs for the same target. In 14 examples suppression of the target mRNA and/or protein levels strongly implies that the drugs are being delivered to the appropriate disease tissues. In a few cases the oligonucleotide drugs have validated their targets.

**Table 1.** Oligonucleotide drugs recently under active development in phase 2 clinical trials or above (Data are extracted from Thomson Reuters Integrity database on 3 April 2012)

	Target	Drug (indication)	References to target inhibition in man
[**1**]	Apolipoprotein B-100 (*APOB*) Non-sense or frameshift mutations in the LDL-receptor binding domain of apoB cause hypercholesterolemia (OMIM 107730)	Mipomersen (pre-registered: FH) 2′-O-MOE ASO administered i.v. or s.c. reduces target protein and LDL cholesterol in serum	(11–14)
[**2**]	Dystrophin (*DMD*) Mutations induce a frame shift or non-sense residue and produces dysfunctional protein (OMIM 300377)	Eteplirsen (phase 2: DMD) Morpholino ASO administered i.v. causes exon skipping and restores dystrophin levels in muscle biopsies	(18)
[**3**]		Drisapersen (phase 3: DMD) 2′-O-Me ASO administered s.c. causes exon skipping and restores dystrophin levels in muscle biopsies of patients	(19)
[**4**]	B cell lymphoma-2 (*BCL2*) Inhibitor of cancer cell apoptosis associated with chemotherapy or radiotherapy resistance	Oblimersen (phase 3: cancer) Phosphorothioate oligodeoxynucleotide ASO administered i.v. continuous infusion inhibits target levels in PBMCs and bone marrow cells	(24–26)
[**5**]	Survivin (*BIRC5*) Inhibitor of cancer cell apoptosis, highly expressed in tumors associated with chemotherapy or radiotherapy resistance	ISIS-23722 (phase 2: cancer) 2′-O-MOE ASO administered i.v. accumulates in tumor tissue, suppresses target, restores apoptosis in tumor cells	(28)
[**6**]	Clusterin (*CLU*) Secreted stress-induced cytoprotective protein, associated with chemotherapy or radiotherapy resistance	Custirsen (phase 3: cancers) 2′-O-MOE ASO administered i.v. suppresses target in prostate cancer tissue and in lymph nodes, increases apoptotic index and improves survival	(32–34)
[**7**]	Heat shock 27 kDa protein 1 (*HSPB1*) Chaperone protein associated with chemotherapy or radiotherapy resistance.	OGX-427 (phase 2: cancer) 2′-O-MOE ASO administered i.v. induces changes in tumor markers, measurable disease and circulating tumor counts.	(38)
[**8**]	Eukaryotic translation initiation factor 4E (*EIF4E*) Protein translation factor	ISIS-EIF4ERx (phase 2: cancer) 2′-O-MOE ASO administered i.v. inhibits eIF-4E mRNA and protein expression, as well as downstream markers in tumor biopsies.	(40)
[**9**]	Ribonucleoside-diphosphate reductase M2 chain (*RRM2*) Essential protein for synthesis of deoxyribonucleotides	GTI-2040 (phase 2: cancer) Phosphorothioate oligodeoxynucleotide ASO administered i.v. with cytarabine in AML patients: reduces RRM2 protein in bone marrow cells in complete responders and not in non-responders	(43, 44)
[**10**]	CCR3, beta chain (*CCR3*, *CSF2RB*) Chemokine signaling proteins mediate eosinophil trafficking in asthma	TPI ASM8 (phase 2: allergic asthma) Combination of two phosphorothioate oligodeoxynucleotide ASOs targeting CCR3 and the beta chain suppress target mRNAs when delivered *via* nebulizer	(48, 49)
[**11**]	ICAM1 (*ICAM1*) Glycoprotein involved in cell trafficking in inflammatory bowel pathophysiology	Alicaforsen (phase 3: pouchitis) Phosphorothioate oligodeoxynucleotide ASO administered i.v. inhibits ICAM-1 protein expression in mucosal biopsies	(51)
[**12**]	C-reactive protein (*CRP*) Elevated levels of CRP are a biomarker of inflammation	ISIS-CRPRx (phase 2: rheumatoid arthritis) 2′-O-MOE ASO reduces levels of CRP by 70% in healthy volunteers	(http://ir.isispharm.com/phoenix.zhtml?c=222170&p=irol-newsArticle_pf&ID=1531705; February 2011)
[**13**]	Transthyretin (*TTR*) Mutations induce formation of aggregated protein deposits (OMIM 176300)	ALN-TTR02 (phase 2: amyloidosis) SiRNA reduces levels of TTR by up to 94% in healthy volunteers. LNP formulated	(http://phx.corporate-ir.net/phoenix.zhtml?c=148005&p=irol-newsArticle_print&ID=1714793&highlight; July 2012)
[**14**]	Mir-122 (*MIR122*) MicroRNA-122 is necessary for translation of HCV genome	Miravirsen (phase 2: hepatitis C) LNA AMO administered s.c. reduces viral RNA in the blood of HCV patients	(56)
[**15**]	TGF-β2 (*TGFB2*)	Trabedersen (phase 3: cancer)	
[**16**]	Nucleocapsid protein (N)	ALN-RSV01 (siRNA) (phase 2: RSV in lung transplant patients)	
[**17**]	Myc (*MYC*)	Resten-CP/Resten-NG (phases 2–3: restenosis)	
[**18**]	Protein kinase B alpha (*Akt1*)	Archexin (phase 2: cancer)	
[**19**]	C-raf kinase (*RAF1*)	iCo-007 (phase 2: diabetic macular edema)	
[**20**]	Spleen tyrosine kinase (*STK*)	Excellair (siRNA) (phase 2: asthma)	
[**21**]	Connective tissue growth factor (*CTGF*)	EXC-001 (phase 2: surgery-related fibrosis)	
[**22**]	Integrin alpha 4 (*ITGA4*)	ATL-1102 (phase 2: multiple sclerosis)	
[**23**]	P53 (*TP53*)	Cenersen (phase 2: cancer)	
[**24**]	P53 (*TP53*)	QPI-1002 (siRNA) (phase 2: prevention of delayed graft function (kidney transplantation).	
[**25**]	DNA damage-inducible transcript 4 (*DDIT4*)	PF-655 (siRNA) (phase 2: diabetic macular edema and macular degeneration)	
[**26**]	Protein tyrosine phosphatase 1B (*PTPN1*)	ISIS-113715 (phase 2: diabetes type 2)	

## RESULTS

**Figure 1.**
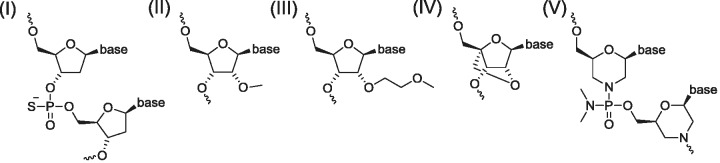
Structures of chemically modified oligonucleotides in Table 1. (**I**) Phosphorothioate oligodeoxyribonucleotide; (**II**) 2′-*O*-methyl-ribonucleotide (2′-*O*-Me); (**III**) 2′-*O*-methoxyethyl (MOE)-ribonucleotide; (**IV**) LNA; and (**V**) Morpholino nucleotide analog.

For this analysis we examined 26 drug/target combinations (Table 1). Most of the drugs are single-stranded, second generation antisense oligonucleotides (ASOs). They are accompanied by five small interfering RNAs (siRNAs) and a single anti-microRNA oligonucleotide (AMO). Most of the ASOs are phosphorothioate oligonucleotides and many have also been chemically modified at selected riboses to enhance their pharmacokinetics (PK) and pharmacodynamics (PD) properties (Figure 1). First generation phosphorothioate oligodeoxynucleotides and second generation oligodeoxynucleotides which are terminally modified with nuclease-resistant, affinity-enhancing modifications induce target mRNA target cleavage *via* RNase H-dependent mechanisms. The remainder modulates splicing, inhibits translation or blocks RNA function (8). To our knowledge none of these drugs is administered to patients using a special formulation.

The targets of these oligonucleotides come from a range of protein families which is notably under-represented in the classical drugable families, i.e. enzymes and receptors. Of the targets, five are enzymes and therefore possibly drugable with small molecules (*PTPN1*, *RRM2*, *SYK*, *RAF1*, *AKT1*). Several are extracellular proteins (*APOB*, *CLU*, *CRP*, *CCR3*/*CSF2RB*, *CTGF*, *VLA-4*, *ICAM1*, *TGFB2, TTR*) and therefore potentially drugable with therapeutic antibodies or proteins. The majority of the targets was clinically unvalidated at the outset of the trials and therefore represents a new mechanism of action for their respective diseases. Three of the targets—*APOB*, *TTR* and *DMD*—are the products of genes which are disregulated or non-functional because of changes in their DNA sequence (e.g. mutations, deletions, translocations, etc.) and which have been proven to cause disease. Hence, they are ‘genetically validated’.

We highlight below 14 drugs for which inhibition of the molecular target at either mRNA or protein level in patient samples has been reported. They are: *APOB*/mipomersen; *DMD*/eteplirsen; *DMD*/drisapersen; *BCL2*/oblimersen; *CLU*/custirsen; *HSPB1*/OGX-427; *BIRC5*/ISIS-23722; *EIF4E*/ISIS-EIF4ERx; *RRM2*/GTI-2040; *ICAM1*/alicaforsen; *CCR3*, *CSF2RB*/TPI ASM8; *CRP*/ISIS-CRPRx; *TTR*/ALN-TTR02; and *MIR122*/miravirsen. The *MIR122*/miravirsen pair is included although miravirsen is not expected to repress levels of the microRNA (miRNA) mir-122. In these 14 cases it can be safely assumed that the oligonucleotide drug is being delivered to its cellular target, although it cannot be assumed that the magnitude of target downregulation is sufficient to mediate a therapeutic effect.

*ApoB/mipomersen for familial hypercholesterolemia (FH)* [***1***]*.* The liver-derived apoliprotein B-100 (ApoB) protein plays a fundamental role in cholesterol homeostasis as a structural component of very low-density lipoprotein (VLDL) and as a ligand for LDL receptor-mediated endocytosis of LDL by liver cells. Its over-production and decreased clearance are seen in cardiovascular-associated diseases, as well as inherited diseases such as familial hypercholesterolemia (FH: OMIM 107730). Familial defective ApoB hypercholesterolemia is a result of non-sense or frame-shift mutations within the LDL-receptor binding domain of ApoB and causes severe hypercholesterolemia and premature cardiovascular disease. Statin drugs are unable to reduce cholesterol sufficiently for these patients. ApoB is an ideal target for an mRNA-targeting oligonucleotide in FH (reviewed in (9)). It is a genetically validated target which is synthesized in the liver, one of the predominant target tissues of ASOs in man. Furthermore, the target protein is secreted into the blood allowing its use as a biomarker to assess directly drug action. Mipomersen is a subcutaneously delivered ASO of 20-nucleotide length (20-mer) and modified with terminal 2′-*O*-methoxyethyl (MOE)-ribonucleotides. It induces ApoB mRNA degradation. It has been tested in numerous clinical trials including phase 3 studies in which efficacy endpoints were met (9,10). The first in-man clinical trial showed rapid, dose-dependent long-term reduction of circulating ApoB of up to 50% (11). In recent published accounts of phase 2 studies, as a single agent and in combination with statins, the drug also showed very efficient target inhibition after treatment of patients with a loading dose and then up to 400 mg/week. This resulted in lowered levels of LDL-cholesterol and triglycerides (up to 71% and 53%, respectively, in one study (12)) constituting clinical validation of the target (12–14). The drug was well tolerated though there have been persistent increases in transaminases, possibly due to pharmacologic inhibition of ApoB (15). It seems highly improbable that mipomersen will face competition for FH from any small-molecule drug if it were to gain approval.

*Dystrophin/eteplirsen, drisapersen for Duchenne muscular dystrophy (DMD)* [***2****,****3***]. Dystrophin is essential for the normal function of skeletal and cardiac muscle. Its gene is prone to mutations that result in muscular dystrophy (muscle weakening) (OMIM: 300377). DMD is mostly due to DNA deletions which disrupt the reading frame and produce a dysfunctional protein. High affinity, chemically modified ASOs can be designed to bind sequence-specifically to pre-mRNA and induce ‘skipping’ of exons containing out-of-frame deletions leading to accumulation of a shortened but nevertheless partially functional protein. This concept was first shown in cells and has been validated in numerous examples *in vivo* (16). The mechanism is now at the heart of a new therapeutic strategy for the treatment of DMD using different sequences of fully modified morpholino (morpholino: eteplirsen) and 2′-*O*-methyl-phosphorothioate RNA (drisapersen) oligonucleotides which induce exon skipping in the dystrophin mRNA. In pioneering clinical trials, treatments of DMD patients with eteplirsen and drisapersen have led to restoration of the functional target protein in high percentages of muscle fibers thereby validating the target (17). For example, in a recently described phase 2 trial young participants received eteplirsen by weekly intravenous infusions of 0.5–20 mg/kg (18). Drug treatments were well tolerated and increases in dystrophin (up to 16% by western blot) and associated proteins were observed in muscle fibers of patients receiving the highest doses of drug. Newly released data from a recent phase 2b trial demonstrated ‘unprecedented’ therapeutic benefits from a once-weekly treatment with eteplirsen at a dose of 50 mg/kg for 36 weeks which resulted in an ∼70 m benefit in standard 6-min functional walk tests (http://investorrelations.sareptatherapeutics.com/phoenix.zhtml?c=64231&p=irol-newsArticle_print&ID=1717599&highlight; July 2012). In a phase 1-2a study of drisapersen treatment of young patients, weekly subcutaneous injections of 0.5–6.0 mg/kg of drug led to new dystrophin expression through exon-51 skipping in all patients receiving the two highest doses. All trial participants then received the highest dose of drug in a 12-week extension phase resulting in a mean improvement of 35 m in the functional walk test (19). Once again, it seems highly improbable that small-molecule drugs could be rationally designed to restore the function of this genetically defective target, though non-selective inducers of ribosomal read-through of premature stop codons have been investigated (reviewed in (17)).

*B-cell leukemia-lymphoma gene 2 (Bcl-2)/oblimersen for cancer* [***4***]*.* Several late-stage oligonucleotide drugs have been or are being investigated for treatment of cancers. Three of the targets—clusterin, survivin, Bcl-2—have well-characterized roles in mechanisms which protect cancer cells from apoptosis induced by cytotoxic drugs. The pro-survival factor Bcl-2 inhibits apoptosis and enhances cell survival by inhibiting the release of cytochrome c during apoptosis. Bcl-2 overexpression in chronic lymphocytic leukemia (CLL) is associated with aggressive disease and resistance to chemotherapy. Bcl-2 family members heterodimerize with pro-apoptotic regulators and efforts to identify small-molecule drugs to inhibit these protein–protein interactions have been largely unsuccessful. One exception is the Bcl-2 antagonist obatoclax mesylate. The drug is in clinical trials however the results to date have not shown strong activity (20). Oblimersen is a first generation 18-mer phosphorothioate oligodeoxynucleotide which potently down-regulates Bcl-2, but also shows an antiproliferative effect through the presence of immunostimulatory CpG motifs in its sequence (21,22). Bcl-2 target downregulation by oblimersen has been demonstrated in humans in several clinical trials (reviewed in (23)). In one, oblimersen was infused into young patients with solid tumors continuously at 7 mg/kg/day for several days in combination with cytotoxics. Reduced Bcl-2 expression was observed in peripheral blood mononuclear cells (PBMCs) in many of the patients before administration of the cytotoxics: in half of these cases reductions were to below 50% of pre-treatment levels (24). In another phase 1 combination trial of oblimersen in acute leukemia patients levels of Bcl-2 transcripts were measured in bone marrow cells of 12 patients after 5 days of treatment, prior to chemotherapy (25). Target downregulation of up to 76% was seen in 75% of the patients and in some of these cases, suppression of Bcl-2 protein of ∼80% was also seen by immunoblotting. In yet another phase 1 study in elderly AML patients administered with oblimersen and cytarabine, or daunorubicin investigators found that those patients with a complete remission (48%) experienced decreased Bcl-2 mRNA and protein levels in bone marrow samples after 72 h of drug infusion and prior to chemotherapy whereas, increased Bcl-2 was seen in the non-responders (26). Clinical trials with oblimersen have spanned more than 10 years. Although some of which have shown survival advantages for patients this drug remains unapproved.

*Survivin/LY2181308 for cancer* [***5***]*.* Survivin is a member of the Inhibitors of apoptosis (IAP) family. In cells, it functions as a stress-induced cytoprotective chaperone exerting its anti-apoptotic effects through binding to the second mitochondrial activator of caspases (SMAC) (reviewed in (27)). It is highly expressed in many human cancer types, but not in differentiated tissues, and is associated with poor prognosis. LY2181308 (ISIS-23722) is an 18-mer ASO modified with terminal 2′-*O*-(MOE) ribonucleotides which targets the translation initiation codon of survivin. An account of a first in-human dose study described how the drug was delivered intravenously as a saline solution initially daily for 3 days as a loading dose and then up to 750 mg weekly into cancer patients. Survivin mRNA and protein in a wide range of tumor tissues were both reduced by ∼20% and the apoptosis marker CC3 indicated restored apoptosis, thereby validating the clinical rationale (28). Positron emission tomography (PET) confirmed that the drug accumulated in solid tumor tissue, as well as kidney and liver (29). No objective responses were seen in these patients and the investigators have speculated that this might be due to an insufficient target downregulation in tumor tissue. The focus now is use of LY2181308 in combination with chemotherapy or radiation. While survivin can be classified as undrugable, in the event of successful development, LY2181308 may face competition from YM155, a small-molecule inhibitor of survivin transcription (30).

*Clusterin/custirsen for cancer* [***6***]*.* Clusterin is a stress-induced, cytoprotective small heat-shock chaperone protein. It is expressed in virtually all tissues and is secreted. In cancer clusterin inhibits apoptosis through its suppression of proapoptotic BAX and activates the PI3K/AKT cell survival pathway (reviewed in (31)). Clusterin levels are increased in a variety of cancers with broad-spectrum resistance to radiation and chemotherapy and inhibition of clusterin enhances apoptosis in xenograft models of cancer. Custirsen (OGX-011) is a 21-mer MOE-modified ASO targeting the clusterin mRNA AUG translation initiation site. The first in-man phase 1 dose-determining study of custirsen was performed in 25 patients with localized prostate cancer using up to 640 mg of intravenously administered ASO, combined with androgen blockade (32). Treatment was well tolerated. Clusterin expression was assayed before and after drug treatment in prostatectomy, as well as in lymph nodes and PBMCs as surrogate tissues. Quantitative reverse transcriptase PCR (Q-PCR) and immunohistochemistry (IHC) showed a very efficient dose dependent inhibition of clusterin mRNA (>90% at the maximum dose) and protein (complete suppression in 57% of cells at the maximum dose) in prostate cancer tissue. This correlated with increasing levels of the drug in prostate cancer tissue measured by ELISA. At the highest dose, the apoptotic index in prostatectomy specimens was increased thereby validating the clinical hypothesis. Downregulation of serum clusterin by ∼40% was documented in a second phase 1 trial (33). This drug progressed into phase 2 clinical trials for various cancers in combination with cytotoxics (34–36). These trials have shown promise for patients in terms of median survival (33) or time to disease progression. Although clusterin is probably undrugable to small-molecule drugs, its validation as a target by custirsen may result in competition from biologics, for example the antibody AB-16B5 or the peptide CGEN-25008.

*Hsp27/OGX-427 for cancer* [***7***]*.* Heat Shock 27 kDa Protein 1 (Hsp27) is another small heat shock protein under clinical investigation as a target for cancer treatment using a MOE-modified ASO. It is highly expressed in several types of cancer: in castration-resistant prostate cancer (CRPC) its expression is induced by androgen removal and/or chemotherapy. It inhibits the activation of caspases as well as activating STAT3 and AKT oncogenic pathways. It is therefore an attractive target in cancer however it is undrugable with small molecules. OGX-427 is a 20-mer MOE ASO which has completed single agent and combination phase 1 trials to determine dose in prostate, bladder, as well as breast and lung cancer. Reduction of tumor markers in prostate (prostate-specific antigen (PSA): >40% reduction in 3 from 16 patients) and ovarian (cancer-antigen 125: >20% reduction in 3 from 5 patients) cancers was reportedly observed in patients, as well as decreased numbers of circulating tumor cells and lowered Hsp27 expression measured by immunofluorescence (37,38).

*EIF-4 E/LY2275796 for cancer* [***8***]*.* The expression of the mRNA cap binding protein eukaryotic initiation factor 4E (EIF-4E) is often upregulated in tumors (39). A 20-mer MOE-ASO (LY2275796) was developed to inhibit EIF-4E and tested in a phase 1 dose finding trial in patients with advanced solid tumors (40). The ASO was administered intravenously in a loading dose over 3 days and then at weekly infusions up to 1000 mg doses. PK parameters were determined from plasma samples using an ELISA to detect the drug. Target downregulation was assessed by comparing EIF-4E mRNA expression and protein IHC from pre- and post-treatment tumor biopsy samples. In 75% of the patients receiving the 1000 mg dose a reduction in target protein expression was seen, and in 6 from 7 cases an 80% inhibition of target mRNA was measured. Two tumor-promoting proteins regulated by EIF-4E—VEGF and cyclin D1—were also reduced. The treatment was well tolerated however no tumor responses were observed at the doses at which target inhibition was achieved. The investigators suggested that the ASO be pursued in combination studies, but at the same time they also cautioned that EIF-4E may not be a valid target.

*RNR/GTI-2040 for cancer* [***9***]*.* Overexpression of ribonucleotide reductase (RNR), an enzyme composed of R1 and R2 subunits which converts ribonucleosides to deoxyribonucleosides, is commonly observed in cancer cells. It increases levels of deoxynucleosides, thereby competing with nucleoside cytotoxics and providing a mechanism of chemoresistance. RNR has been validated as a target in cancer using small-molecule drugs however selectivity issues with such compounds have been reported (41,42). Increased cellular levels of RNR reduce the effectiveness of nucleoside anticancer drugs such as cytarabine (AraC), a cytidine derivative which is incorporated into and halts DNA synthesis (see references in (43)). GTI-2040 is a first generation 20-mer phosphorothioate oligodeoxynucleotide which inhibits the R2 subunit (RRM2). This creates an imbalance in cellular levels of cytidine triphosphate and favors incorporation of AraC into DNA. GTI-2040 has been tested in a handful of phase 1 and phase 1/2 trials, as a single agent and in combination. As with other phosphorothioate oligodeoxynucleotides the drug has a short plasma half-life and is continuously infused into patients over several days. Target downregulation was seen in a phase 2 trial of GTI-2040 in white blood cells and tumor tissue assayed from a single patient before and after drug treatment (44). A strong suppression (25-fold decrease) of the target mRNA was seen providing confirmation of delivery of the drug to tumor tissue. In a dose escalation phase 1 trial, patients with acute myeloid leukemia (AML) were infused intravenously with GTI-2040 prior to exposure to high doses of AraC (43). Levels of GTI-2040 were measured in plasma and the lysates of bone marrow cells after initiation of infusion. R2 protein expression was measured pre- and post-treatment in bone marrow cells at the same time-points. A trend for preferred uptake of oligonucleotide into leukemic cells was observed and drug concentrations in cells increased 2-fold over the measurement time-period. Furthermore, reductions of R2 protein to >50% were seen in the bone marrow samples from some patients. Complete remission was associated with a significant reduction in target protein levels, whereas non-responders showed increased levels. A phase 2 study of GTI-2040 in combination with docetaxel and prednisone in CRPC was less encouraging (45): continuous infusion over 14 days of drug led to a PSA response in less than half of the patients. The trial was not extended with the investigators citing insufficient dosing to achieve target downregulation, difficulties to deliver intravenous dosing for 14 continuous days and doubts concerning the relevance of the target for CRPC.

*CCR3, β_c_ chain/TPI ASM8 for asthma* [***10***]*.* Eosinophils play an important pathogenic role in allergic asthma. The late asthmatic response (LAR) to inhaled allergen challenge induces the proliferation and migration of eosinophils. This depends upon chemokine signaling involving C-C chemokine receptor type 3 (CCR3) and the stimulation of receptors for the cytokines IL-3, IL-5 and granulocyte-macrophage colony-stimulating factor (GM-CSF) comprising a common beta chain (*β*_c_). Indeed, IL-5 has been proposed as an important drug target for allergy and associated disorders (reviewed in (46)) and at least two antibodies against the IL-5 cytokine and its receptor are in clinical testing. The drug product TPI ASM8 comprises two first generation 19-mer phosphorothioate oligodeoxynucleotides (adenines in the sequence are replaced with 2′-amino-2′-deoxyadenine) directed to CCR3 and the common *β*_c_. By targeting multiple factors possibly playing redundant roles in the disease, a highly effective response was anticipated in patients. In one clinical trial, patients with mild atopic asthma were treated by nebulizer with 1.5 mg per day of drug over 4 days (47). A significant reduction in early asthmatic response (EAR) and a trend for reduced LAR were reported. Target mRNA inhibition was monitored in sputum samples. Drug uptake into the cells was monitored by hybridization ELISA. TPI ASM8 inhibited the increase in sputum eosinophils induced by the allergen. In six of the eight patients assayed, target mRNA levels were lowered. Although suppressed cell surface protein of CCR3 or *β*_c_ was not confirmed, the authors suggest that this may be due to analysis of inappropriate cell types. Results of a second trial have since emerged with similar results (48,49). Drug treatment was safe and well tolerated: dose response was reported and efficacy was maintained with one administration per day suggesting clinical validation of the target and showing promise for the drug.

*ICAM1/alicaforsen for pouchitis* [***11***]*.* Intercellular adhesion molecule-1 (ICAM1) is a transmembrane glycoprotein expressed in endothelial cells and leukocytes. It facilitates leukocyte migration from blood to sites of inflammation and is involved in inflammatory bowel pathophysiology. Inflammatory bowel disease (IBD) encompasses an array of disorders including Crohn’s disease (CD) and ulcerative colitis (UC). ICAM1 was selected as a target for IBD because of its role in cell trafficking, because it is present during intestinal inflammation and because increasing levels of circulating protein are observed in patients with CD, UC and pouchitis. Alicaforsen is a 20-mer phosphorothioate oligodeoxynucleotide targeted to ICAM1. Several clinical trials of alicaforsen have been completed (reviewed in (50)). Clinical trials of intravenously delivered drug in patients with CD failed to show a significant effect on disease, although intestinal biopsy samples from patients in one of the trials showed reduced levels of intestinal mucosal ICAM1 expression in the majority of the patients given the highest 2 mg/kg drug dose (51). It has since been suggested that some of the patients selected for these CD trials did not in fact have CD, but rather irritable bowel syndrome, contributing to the failed trial outcomes (52). Alicaforsen was delivered as an enema for UC in larger clinical trials and has demonstrated some reduction in disease activity in the long term (53), but the most promising data have been obtained in treatment of pouchitis. In a small open-label trial of alicaforsen the drug was shown to be safe and well tolerated (54). The results from nightly enema formulations of up to 240 mg administered for 6-weeks were evaluated by endoscopy during the treatment and 4 weeks following treatment. Overall a statistically significant reduction in pouchitis disease activity index was observed and 58% of patients were in remission, and trials were considered very promising (reviewed in (50)). Interestingly, longer-term benefits were unexpectedly observed in many patients and the investigators have called for further trials not only to evaluate alicaforsen as a drug, but also to validate ICAM1 as an effective target in the inflammatory pathway.

*C-Reactive protein/ISIS-CRPRx for cardiovascular disorder* [***12***]*.* C-reactive protein (CRP) is produced in the liver and increased levels of the protein are seen in a variety of disorders including cardiovascular disease, diabetes and various inflammatory conditions. ISIS-CRPRx is a MOE ASO which recently completed a phase 1 clinical trial in 80 subjects at single and multiple doses from 50–600 mg/week (http://ir.isispharm.com/phoenix.zhtml?c=222170&p=irol-newsArticle_pf&ID=1531705; February 2011). An average reduction of more than 70% in circulating CRP was reported. The drug sponsors have announced that this first CRP inhibitor will be employed as an early proof of concept for the target in a variety of diseases, including multiple myeloma and rheumatoid arthritis.

*TTR/ALN-TTR02 for amyloidosis [****13****].* We identified five siRNAs in phase 2 clinical trials ([**13**], [**16**], [**20**], [**24**], [**25**]). From these, target suppression has only been reported for one—ALN-TTR02, though a working RNAi mechanism in man was shown for a formulated siRNA whose development has since been discontinued (55). The hereditary form of *amyloidosis,* ATTR (OMIM: 176300) is caused by mutations in the transthyretin gene (*TTR*), which is expressed predominantly in the liver, and results in the accumulation of pathogenic deposits of mutant and wild-type TTR protein in multiple extra-hepatic tissues, including the peripheral nervous system and heart. The intravenously administered, lipid nanoparticle (LNP)-formulated chemically unmodified siRNA ALN-TTR02 targets the mutant TTR protein responsible for TTR-mediated amyloidosis**.** Recently this siRNA has progressed into phase 2 trials following promising phase 1 results in which the drug was well tolerated and demonstrated an up to 94% reduction in serum TTR levels in healthy volunteers after a single dose (0.01–0.50 mg/kg) (http://phx.corporate-ir .net/phoenix.zhtml?c=148005&p=irol-newsArticle_print&ID=1714793&highlight; July 2012). Furthermore, TTR downregulation was reportedly sustained at up to 80% 1 month after treatment ceased.

*Miscellaneous drug/target combinations not reporting target inhibition.* Many of the oligonucleotide drugs in advanced clinical trials reportedly have positive effects in patients but, for a variety of possible reasons, the PD data describing inhibition of target by the drug has not been reported (Table 1, [**14–26**]). Miravirsen (SPC-3649: (**14**)) is a 15-mer locked nucleic acid (LNA)-modified phosphorothioate oligodeoxynucleotide ASO. The LNA modification, interspersed throughout an oligodeoxyribonucleotide sequence, endows the molecule with nuclease stability and high affinity for its target, the host liver-specific miRNA mir-122, which it sequesters in a duplex structure thereby blocking its function without inducing its degradation. Mir-122 is highly conserved across several species and regulates lipid metabolism. However, it also interacts at adjacent sites in the 5′ terminus of the hepatitis C viral (HCV) RNA, resulting in increased viral RNA abundance by a poorly understood mechanism. Miravirsen has shown long-lasting suppression of HCV viremia in chronically infected chimpanzees and is the first anti-miRNA drug to enter the clinic. Weekly subcutaneous injections of the drug at up to 7 mg/kg have strongly reduced HCV RNA in patients, in one case to undetectable levels after 1 month (56). Circulating levels of cholesterol, ApoA and ApoB have served as efficacy biomarkers for the drug.

Trabedersen (AP12009: [**15**]) is a 20-mer first generation phosphorothioate oligodeoxynucleotide targeting TGF-β2 mRNA in phase 3 clinical trials. The drug has generated promising data in numerous clinical trials for the treatment of malignant brain cancers after local delivery directly into tumors (reviewed in (57)) however, correlation of the anti-tumor activity with target downregulation has not yet been reported (58).

To date, target inhibition has not been reported for the majority of the siRNAs in clinical trials, partly reflecting the younger status of this field. One of the most clinically advanced siRNAs is the intranasally administered siRNA ALN-RSV-01 [16] which targets the nucleocapsid protein of respiratory syncytial virus (RSV), a structural protein responsible for encapsidation of the viral genome. This siRNA is an unmodified 19-mer RNA phosphorodiester duplex with 3′-overhangs of thymidine deoxynucleotides. In phase 1 trials it proved to be safe and well tolerated in healthy subjects (59) and in experimentally infected volunteers ALN-RSV-01 treatment prevented RSV infection (60). In lung transplant patients with RSV infections it reduced symptoms and the incidence of new or progressive bronchiolitis obliterans syndrome caused by the infections (61). A larger trial is ongoing.

## DISCUSSION

Properly designed oligonucleotides are specific inhibitors of gene expression. They are well suited to the modern paradigm of pharmaceutical research which begins with the gene coding for a disease-relevant target. Where a target represents a new mechanism of action and is being investigated for the first time in patients there is a steep learning curve and a high probability of failure (6). We examined 26 oligonucleotide drug/target combinations which are, or were recently, in phase 2 clinical trials or above according to the Thomson Reuters Integrity database (Table 1). In at least 14 cases downregulation of target mRNA or protein levels in the relevant tissues/cell types in patients has been described in publications or company web-releases, confirming that the drugs were able to reach their targets in liver, muscle fibers, various solid tumors, lymph nodes, bone marrow, eosinophil progenitors, blood cells and intestinal mucosa. Thirteen of these cases involved single-stranded oligonucleotides, and in none was a formulation used to enhance delivery. The sole siRNA in the group was administered in a LNP-formulation.

For the less metabolically stable, first generation phosphorothioate oligodeoxyribonucleotides local administration has been a favored form of delivery. The first therapeutic ASO to be approved, fomiversen, was a full phosphorothioate oligodeoxyribonucleotide targeting the viral *IE2* gene of human cytomegalovirus which was administered by intravitreal injection (62). A local delivery was also employed for four of the drugs discussed above: TPI ASM8 (pulmonary administration), alicaforsen (enema), trabedersen (intracranial infusion) and the siRNA ALN-RSV-01 (pulmonary administration). In the remainder of the cases the drug is delivered intravenously by infusion or subcutaneously. For the first generation phosphorothioate oligodeoxyribonucleotides GTI-2040 and oblimersen the drug infusions continued over many days, in some cases causing technical difficulties (45). For the second generation molecules (MOE, morpholino, LNA) it was sufficient to administer drug doses weekly. This may reflect greater serum stability of the second generation molecules which, together with their added potency, is a tribute to extensive research on modification of the ribose by medicinal chemists (63–66).

*In vivo*, drugs need to cross numerous biological barriers to reach their targets (reviewed in (8,67)). After systemic administration single-stranded phosphorothioate oligonucleotides circulate non-covalently bound to plasma proteins which, because of their size, slow down their renal filtration and clearance. In this form they are also protected from nuclease degradation. They pass across the vascular endothelial barrier and diffuse through the extracellular matrix to distribute broadly in the body, particularly to liver, adipose tissue, spleen, kidney, bone, bone marrow, intestine and macrophages. The oligonucleotides transverse plasma membranes of cells possibly by receptor-mediated endocytosis to reach the cytoplasm in vesicles, though receptors responsible for cellular entry have not been characterized. Intracellular trafficking into endosomes and/or lysosomes follows, and the oligonucleotides can escape in small amounts from some of these compartments in order to access their RNA targets in the cytoplasm and the nucleus. The mechanisms of intracellular trafficking are poorly understood and are the subject of ongoing research. Strong pharmacokinetic/pharmacodynamic (PK/PD) relationships—where the distribution of the drug correlates with its observed activity—have been established in the liver and the kidney cortex in mice for both phosphorothioate oligodeoxynucleotides and MOE-modified oligonucleotides (68). However, clinical PK/PD relationships require predictive PK and reliable PD biomarkers, therefore comparatively few PK/PD relationships for oligonucleotide drugs in man have been described. From the drugs highlighted above, strong clinical PD/PK relationships were demonstrated for custirsen in prostate cancer and for mipomersen.

Target selection is a critical phase in drug discovery, particularly for oligonucleotide drugs. Decreasing productivity in pharma R&D has cast doubts on the modern paradigm of pharmaceutical research that begins with a target gene: many successful drugs originally discovered by compound profiling modulate multiple targets and it is recognized that drug targets often have redundancy pathways (69), limiting the choice of targets for a gene-specific approach. In addition, it is preferable that oligonucleotide drugs do not face competition with cheaper mainstream drugs for the same target. This is achieved by working outside of the drugable genome on novel mechanisms; however, the risk of project failure is increased.

Most of the 26 targets of Table 1 represent new mechanistic targets. Three of the targets from Table 1 (*APOB, DMD, TTR*) are genetically validated: mutations in the DNA sequence of the gene cause the disease. For *APOB* and *DMD*, second generation ASOs (mipomersen, eteplirsen, drisapersen) have shown the expected effects on the target protein in patients in multiple clinical trials. In turn, this has yielded improvements in disease thereby providing clinical validation of the targets. A subset of the targets (*BCL2*, *CLU*, *HSPB1*, *BIRC5*, *EIF4E* and *RRM2*) is being investigated for cancers in combination with approved cytotoxic drugs. Varying degrees of target mRNA and protein inhibition by oligonucleotides have been observed in tumor tissues in patients and in many cases this has been associated with anti-tumor activity, However, the general lack of robust effects on disease outcome seen so far leads some investigators to question whether it is insufficient target inhibition due to sub-optimal drug delivery or an invalid target which is to blame (40,70). As small-molecule inhibitors of several of these targets (obatoclax/BCL2 (20), gemcitabine/RNR (45) and YM155/survivin (30)) have also yet to be successful, the validity of the targets may indeed be suspect. It is to be expected that many of the drugs described here will eventually be withdrawn from development. Although detailed information concerning the reasons for failure is often not publicly available, poor drug tolerability and insufficient target inhibition will likely be responsible for some of these cases, traits that are often associated with use of first generation phosphorothioate oligodeoxynucleotides. In other cases however, as we emphasize here, inefficient target delivery, inappropriate disease target selection, sub-optimal trial design and patient selection may all contribute to unsuccessful outcomes.

Oligonucleotide drugs still represent a promising new paradigm in drug discovery/development. Emerging data have confirmed that they are delivered to many organs/tissues at concentrations sufficient to inhibit their RNA targets and see therapeutic effects in man. New robust methods of, for example, LNP-formulated delivery to currently inaccessible tissues may expand their application further, once associated issues of toxicity, manufacturing complexity and cost have been solved. Although in principle, oligonucleotides can be targeted to any gene of the genome, in reality, they will likely be most successful when targeting genes in new mechanisms outside of the drugable genome and in monogenic diseases.

## FUNDING

Novartis Foundation (formerly the Ciba Geigy Jubilee Foundation) (to H.L.L.). Funding for open access charge: ETH Zürich, Zürich.

*Conflict of interest statement*. None declared.
